# Supplementary material for: Associations of Anti-Aquaporin 5 Autoantibodies with Serologic and Histopathological Features of Sjögren’s Syndrome
Source: J Clin Med. 2019 Nov 3;8(11):1863. doi: 10.3390/jcm8111863 (PMC6912604; doi:10.3390/jcm8111863)

Supplementary Table S1. Bivariate analysis exploring the presence of anti-AQP5 autoantibodies by disease criteria for Sjogren's syndrome in 154 SICCA registry samples.

Disease criteria for Sjogren's syndrome		n	anti-AQP5 IgA by CB-IFC			anti-AQP5_C2 IgG by CB-IFC			anti-AQP5_ A IgG by ELISA			anti-AQP5_C2 IgG by ELISA			anti-AQP5_AC2E1 IgG by ELISA		
			≥ 0.83 (au)	< 0.83 (au)	*p**	≥ 1.79 (au)	< 1.79 (au)	*p**	≥ 54.1 (ng/ml)	< 54.1 (ng/ml)	*p**	≥ 44.3 (ng/ml)	< 44.3 (ng/ml)	*p**	≥ 34.9 (ng/ml)	< 34.9 (ng/ml)	*p**
Serum anti-SSA	Positive	102	**42 (41.2%)**	**60 (58.8%)**	**<0.001**	**62 (60.8%)**	**40 (39.2%)**	**0.004**	**73 (71.6%)**	**29 (28.4%)**	**<0.001**	**64 (62.7%)**	**38 (37.3%)**	**0.001**	**84 (82.4%)**	**18 (17.6%)**	**<0.001**
	Negative	52	**4 (7.7%)**	**48 (92.3%)**		**19 (36.5%)**	**33 (63.5%)**		**21 (40.4%)**	**31 (59.6%)**		**18 (34.6%)**	**34 (65.4%)**		**26 (50.0%)**	**26 (50.0%)**	
Serum anti-SSB	Positive	61	23 (37.7%)	38 (62.3%)	0.085	36 (59.0%)	25 (41.0%)	0.196	43 (70.5%)	18 (29.5%)	0.051	35 (57.4%)	26 (42.6%)	0.483	**51 (83.6%)**	**10 (16.4%)**	**0.007**
	Negative	93	23 (24.7%)	70 (75.3%)		45 (48.4%)	48 (51.6%)		51 (54.8%)	42 (45.2%)		48 (51.6%)	45 (48.4%)		**59 (63.4%)**	**34 (36.6%)**	
RF	Positive	75	**29 (38.7%)**	**46 (61.3%)**	**0.02**	**47 (62.7%)**	**28 (37.3%)**	**0.015**	**54 (72.0%)**	**21 (28.0%)**	**0.007**	**47 (62.7%)**	**28 (37.3%)**	**0.022**	**64 (85.3%)**	**11 (14.7%)**	**<0.001**
	Negative	79	**17 (21.5%)**	**62 (78.5%)**		**34 (43.0%)**	**45 (57.0%)**		**40 (50.6%)**	**39 (49.4%)**		**35 (44.3%)**	**44 (55.7%)**		**46 (58.2%)**	**33 (41.8%)**	
ANA	Positive	75	**33 (44.0%)**	**42 (56.0%)**	**<0.001**	**47 (62.7%)**	**28 (37.3%)**	**0.015**	**53 (70.7%)**	**22 (29.3%)**	**0.017**	**52 (69.3%)**	**23 (30.7%)**	**<0.001**	**67 (89.3%)**	**8 (10.7%)**	**<0.001**
	Negative	69	**13 (18.8%)**	**66 (95.7%)**		**34 (49.3%)**	**45 (65.2%)**		**41 (59.4%)**	**38(55.1%)**		**30 (43.5%)**	**49 (71.0%)**		**43 (62.3%)**	**36 (52.2%)**	
UWSFR	≤ 0.1ml/min	99	30 (30.3%)	69 (69.7%)	0.875	57 (57.6%)	42(42.4%)	0.097	60 (60.6%)	39 (39.4%)	0.883	54 (54.5%)	45 (45.5%)	0.828	70 (70.7%)	29 (29.3%)	0.790
	> 0.1ml/min	55	16 (29.1%)	39 (70.9%)		24 (43.6%)	31 (56.4%)		34 (61.8%)	21 (38.2%)		29 (52.7%)	26 (47.3%)		40 (72.7%)	15 (27.3%)	
FLS score	≥ 1	80	**29 (36.3%)**	**51 (63.8%)**		**50 (62.5%)**	**30 (37.5%)**		**57 (71.3%)**	**23 (28.8%)**		**53 (66.3%)**	**27 (33.8%)**		**67 (83.8%)**	**13 (16.3%)**	
	0 << 1	15	**5 (33.3%)**	**10 (66.7%)**	**0.029**	**5 (33.3%)**	**10 (66.7%)**	**0.008**	**9 (60.0%)**	**6 (40.0%)**	**0.002**	**5 (33.3%)**	**10 (66.7%)**	**0.002**	**10 (66.7%)**	**5 (33.3%)**	**<0.001**
	= 0	48	**7 (14.6%)**	**41 (85.4%)**		**18 (37.5%)**	**30 (62.5%)**		**19 (39.6%)**	**29 (60.4%)**		**18 (37.5%)**	**30 (62.5%)**		**23 (47.9%)**	**25 (52.1%)**	
Schirmer's test	< 5mm in 5min	74	47 (63.5%)	27 (36.5%)	0.579	41 (55.4%)	33 (44.6%)	0.480	47 (63.5%)	27 (36.5%)	0.579	43 (58.1%)	31 (41.9%)	0.361	57 (77.0%)	17 (23.0%)	0.252
	≥ 5mm in 5min	79	46 (58.2%)	33 (41.8%)		39 (49.4%)	40 (50.6%)		46 (58.2%)	33 (41.8%)		39 (49.4%)	40 (50.6%)		52 (65.8%)	27 (34.2%)	
Ocular staining score	≥ 3	105	**42 (40.0%)**	**63 (60.0%)**	**<0.001**	**66 (62.9%)**	**39 (37.1%)**	**<0.001**	**73 (69.5%)**	**32 (30.5%)**	**0.002**	**65 (61.9%)**	**40 (38.1%)**	**0.002**	**85 (81.0%)**	**20 (19.0%)**	**<0.001**
	< 3	49	**4 (8.2%)**	**45 (91.8%)**		**15 (30.6%)**	**34 (69.4%)**		**21 (42.9%)**	**28 (57.1%)**		**17 (34.7%)**	**32 (65.3%)**		**25 (51.0%)**	**24 (49.0%)**	

SSA: Sjögren's syndrome-related antigen A; SSB: Sjögren's syndrome-related antigen B; RF: Rheumatoid factor; ANA: anti-nuclear antibody; UWSFR: Unstimulated whole salivary flow rate; FLS: Focal lymphocytic sialadenitis; au: arbitrary unit. *By either chi-square or Fisher's exact test. Bold denotes statistical significance at $p < 0.05$.

Supplementary Table S2. Spearman's rank correlation among the levels of anti-AQP5 autoantibodies

				CB-IFC					ELISA							
				IgA	IgG				IgG				IgA			
Method	Isotype	Ag		AQP5	A	C2	E1	AQP5	A	C2	E1	AC2E1	A	C2	E1	AC2E1
CB-IFC	IgA	AQP5	*r*	1.000	0.063	0.139	**0.298**	**0.202**	-0.055	-0.031	0.055	-0.060	-0.059	0.007	-0.047	-0.058
			P		0.438	0.085	**0.000**	**0.012**	0.500	0.704	0.500	0.456	0.465	0.927	0.560	0.478
	IgG	A	*r*		1.000	**0.618**	**0.553**	**0.579**	0.083	0.142	0.020	0.143	0.088	0.059	**0.185**	0.139
			P			**0.000**	**0.000**	**0.000**	0.308	0.078	0.803	0.078	0.278	0.464	**0.022**	0.086
		C2	*r*			1.000	**0.652**	**0.558**	0.042	0.034	0.033	0.094	0.116	0.054	**0.205**	0.133
			P				**0.000**	**0.000**	0.607	0.672	0.682	0.244	0.152	0.503	**0.011**	0.100
		E1	*r*				1.000	**0.686**	0.083	0.031	0.039	0.117	0.050	0.076	0.049	0.104
			P					**0.000**	0.304	0.707	0.635	0.149	0.541	0.350	0.549	0.200
		AQP5	*r*					1.000	0.014	0.049	-0.016	0.082	0.069	0.080	0.033	0.081
			P						0.864	0.544	0.848	0.313	0.398	0.324	0.687	0.317
ELISA	IgG	A	*r*						1.000	**0.535**	**0.468**	**0.793**	**0.255**	**0.204**	0.104	**0.179**
			P							**0.000**	**0.000**	**0.000**	**0.001**	**0.011**	0.200	**0.026**
		C2	*r*							1.000	**0.738**	**0.641**	0.121	**0.244**	0.159	**0.212**
			P								**0.000**	**0.000**	0.135	**0.002**	0.049	**0.008**
		E1	*r*								1.000	**0.619**	0.078	**0.167**	0.130	0.147
			P									**0.000**	0.336	**0.038**	0.109	0.070
		AC2E1	*r*									1.000	**0.274**	**0.258**	0.121	**0.218**
			P										**0.001**	**0.001**	0.135	**0.007**
	IgA	A	*r*										1.000	**0.345**	**0.362**	**0.696**
			P											**0.000**	**0.000**	**0.000**
		C2	*r*											1.000	**0.503**	**0.482**
			P												**0.000**	**0.000**
		E1	*r*												1.000	**0.652**
			P													**0.000**
		AC2E1	*r*													1.000
			P													

Bold denotes statistical significance at $p < 0.05$.

Supplementary Table S3. Bivariate analysis exploring the presence of anti-AQP5 IgG by other autoantibodies in SLE and RA samples.

			anti-AQP5_ A IgG			anti-AQP5_C2 IgG			anti-AQP5_E1 IgG			anti-AQP5_AC2E1 IgG		
		n	≥ 54.1 (ng/ml)	< 54.1 (ng/ml)	p^*	≥ 44.3 (ng/ml)	< 44.3 (ng/ml)	p^*	≥ 19.3 (ng/ml)	<19.3 (ng/ml)	p^*	≥ 34.9 (ng/ml)	< 34.9 (ng/ml)	p^*
SSA in SLE	Positive	20	2 (10.0%)	18 (90.0%)	0.673	4 (20.0%)	16 (80.0%)	0.468	5 (25.0%)	15 (75.0%)	0.276	9 (45.0%)	11 (55.0%)	0.254
	Negative	9	2 (22.2%)	7 (77.8%)		2 (22.2%)	7 (77.8%)		5 (55.6%)	4 (44.4%)		7 (77.8%)	2 (22.2%)	
ANA in RA	Positive	14	2 (14.3%)	12 (85.7%)	0.169	1 (7.1%)	13 (92.9%)	0.862	7 (50.0%)	7 (50.0%)	0.985	5 (35.7%)	9 (64.3%)	0.111
	Negative	17	0 (0.0%)	17 (100%)		1 (5.9%)	16 (94.1%)		8 (47.1%)	9 (52.9%)		12 (70.6%)	5 (29.4%)	
RF in RA	Positive	27	3 (11.1%)	24 (88.9%)	0.447	2 (7.4%)	25 (92.6%)	0.590	14 (51.9%)	13 (48.1%)	0.380	16 (59.3%)	11 (40.7%)	0.473
	Negative	8	0 (0.0%)	8 (100%)		0 (0.0%)	8 (100%)		3 (37.5%)	5 (62.5%)		4 (50.0%)	4 (50.0%)	

SSA: Sjögren's syndrome-related antigen A; ANA: anti-nuclear antibody; RF: Rheumatoid factor; *By either chi-square or Fisher's exact test.

Supplementary Figure S1

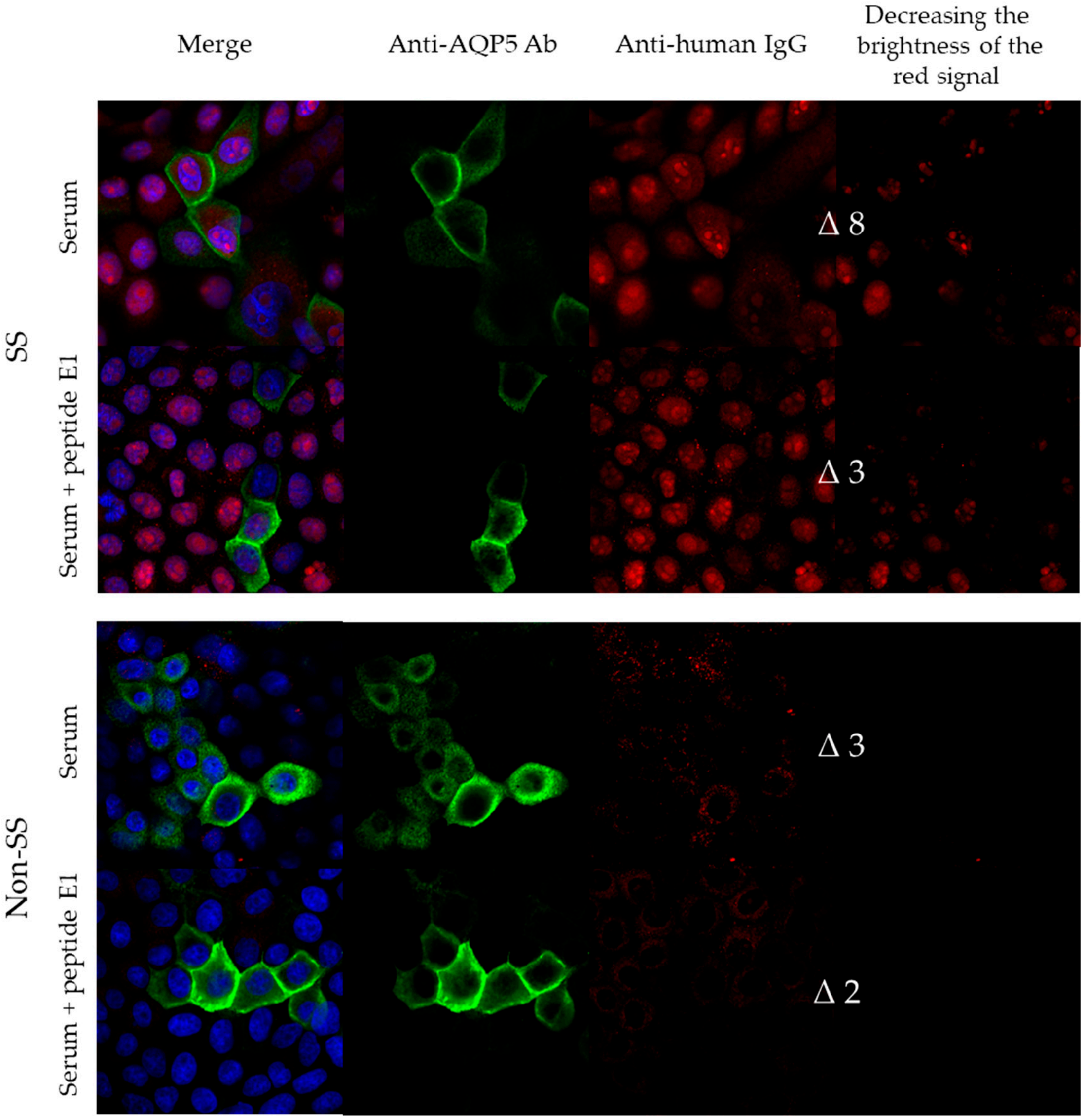

Supplement: Supplementary file 1 [file jcm-08-01863-s001.pdf]
